# The Value of Effective Lung Ventilation Area Ratio Based on CT Image Analysis Is a New Index to Predict the Shorter Outcome of Anti-melanoma Differentiation-Associated Protein 5 Positive Dermatomyositis Associated Interstitial Lung Disease: A Single-Center Retrospective Study

**DOI:** 10.3389/fmed.2021.728487

**Published:** 2021-10-07

**Authors:** Changjian Wang, Jinfeng Du, Xilong Mei, Lingchao Guo, Fangzhao Li, Hong Luo, Fen Li

**Affiliations:** ^1^College of Computer, National University of Defense Technology, Changsha, China; ^2^Department of Rheumatology and Immunology, The Second Xiangya Hospital of Central South University, Changsha, China; ^3^Department of Radiology, The Second Xiangya Hospital of Central South University, Changsha, China; ^4^Department of Respiratory and Critical Care Medicine, The Second Xiangya Hospital of Central South University, Changsha, China

**Keywords:** dermatomyositis, anti-melanoma differentiation-associated protein 5 antibody, interstitial lung disease, prognosis, effective lung ventilation area ratio, computer-aided image analysis

## Abstract

**Background:** Anti-melanoma differentiation-associated protein 5 (MDA5) positive dermatomyositis (MDA5^+^DM) patients have poor outcomes due to rapidly progressive interstitial lung disease (ILD). The accurate assessment of lung involvement is an urgent focus of research.

**Methods:** A computer-aided lung interstitial image analysis technology has been developed, and a quantitative indicator named effective lung ventilation area ratio (ELVAR) that calculates the proportion of the area outside the lung interstitium in lung tissue has been established. 55 newly diagnosed MDA5^+^DM patients and 46 healthy individuals, matched for age and gender, were enrolled in this study. MDA5^+^DM patients were classified into early death group or early survival group according to their survival state within 3 months after diagnosis. Clinical characteristics, laboratory and immunological test results, lung involvement (including ELVAR value) and treatment were compared between early death group and early survival group to determine an index that can predict prognoses of patients with MDA5^+^DM.

**Results:** There were significant differences between early death MDA5^+^DM patients and early survival MDA5^+^DM patients about 12 indices including age of onset, CRP, ferritin, albumin, and pulmonary involvement including severity of type I respiratory failure at diagnosis, P/F ratio, oxygen supplementation, values of ELVAR, FVC, and DLCO. The results of ROC analysis and correlation analysis showed the value of ELVAR had good diagnostic value and widely correlation with many clinical characteristics. Univariate analysis and Multivariate analysis showed four factors including age of onset, ferritin, value of ELVAR, and oxygen supplementation >4 L/min significantly value for poor prognosis in MDA5^+^DM patients. A cutoff value of 0.835 about ELVAR had good predictive power for mortality within 3 months in 54.2% of MDA5^+^DM patients.

**Conclusion:** The value of ELVAR derived from computed tomography image analysis is a new index that can predict poor outcomes in MDA5^+^DM patients with rapidly progressive interstitial lung disease.

## Introduction

Idiopathic inflammatory myopathy (IIM) are heterogenous family of diseases (1). Dermatomyositis (DM) is a subtype of IIM that is characterized by skin rash and myopathy. The incidence of DM in the American population is approximately 1–6 per 100,000 people (2). Although the cause of DM is unknown, recent studies have revealed that anti-melanoma differentiation-associated protein 5 (MDA5) is associated with a subtype of DM (3). MDA5 promotes the production and activation of type I interferon (IFN), which is involved in the pathogenesis of DM (4, 5). Several clinical studies have found that MDA5^+^DM patients are more likely to develop rapidly progressive interstitial pneumonia (ILD) with life-threatening characteristics (6–9). Even after receiving active drug therapy, MDA5^+^DM patients may die soon after, and 6-months mortality has been reported to be as high as 59% (10, 11). Therefore, the accurate assessment of prognoses of MDA5^+^DM patients is an urgent focus of research.

The advantage of high-resolution computed tomography (HRCT) is its ability to detect interstitial pneumonia with mild lesions in the early stage of the disease. The current limitation is the lack of a quantitative method to accurately identify subtle changes in abnormalities. Although several studies on the quantitative analysis of lung interstitial lesions in patients and mice have been conducted (12–16), results have not been convincing. Our study aimed to develop metric tools for clinicians to accurately assess the severity of interstitial pneumonia and establish a methodological plat that combines radiology and computer technology to predict prognoses of patients with MDA5^+^DM.

## Materials and Methods

### Patient Data Collection

**Figure 1:**
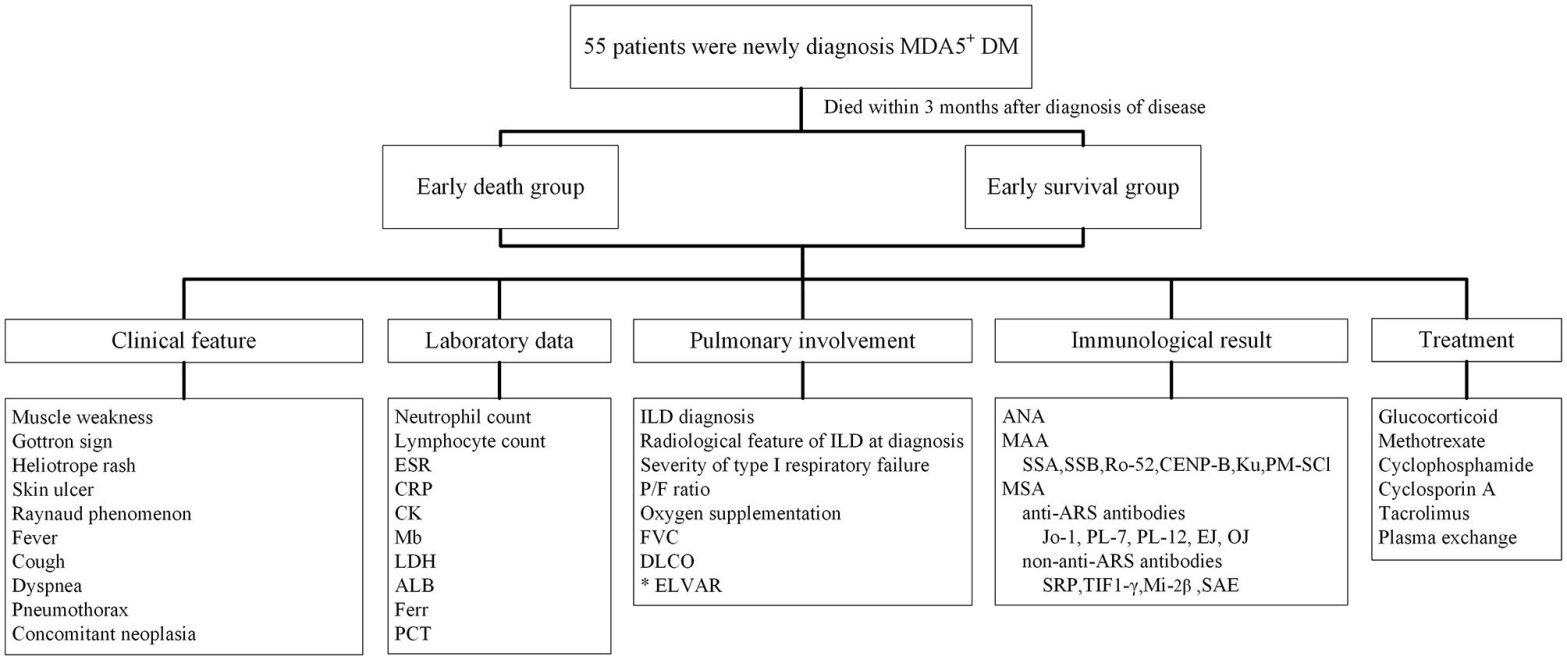
Flow chart of clinical research design for newly diagnosed MDA5^+^DM patients. ALB, albumin; ANA, antinuclear antibody; ARS, minoacyl-tRNA synthetase; CRP, C reactive protein; CK, creatinine kinase; DLCO, carbon monoxide diffusing capacity; ELVAR, effective lung ventilation area ratio; ESR, erythrocyte sedimentation rate; Ferr, ferritin; FVC, forced vital capacity; ILD, interstitial lung disease; LDH, lactate dehydrogenase; MAA, myositis associated antibody; Mb, Myoglobin; MDA5, melanoma melanoma differentiation associated gene 5; MSA, myositis specific antibody; PCT, procalcitonin; P/F ratio, PaO2/FiO2 ratio; SRP, signal recognition particle; SAE, anti-small ubiquitin-like modifier antibody; TIF1-γ, transcription intermediary factor 1-gamma.

This single-center retrospective study was approved by the ethics committee of the Second Xiangya Hospital of Central South University (approval number: MDA5-IIM2020). All 55 MDA5^+^DM patients were newly onset and newly diagnosed fulfilling expert consensus from 239th ENMC International Workshop with MDA5 positive characteristic (17). Patients were hospitalized in the ward of Department of Rheumatology and Immunology between January 1st, 2015 and December 31st, 2018. Those who died within 3 months after diagnosis were classified into early death group, and those who had survived more than 3 months after diagnosis were classified into early survival group. Patient survival status was confirmed by medical records or by telephone. The survival status of patients in early survival group was verified 1 year later by telephone. Clinical characteristics, laboratory data, pulmonary involvement, antibody positive, and treatment information were obtained retrospectively from medical records. ILD diagnoses were verified by a radiologist and a respirologist (Figure 1). As control group, 46 healthy subjects, matched for age and gender, were included in the study to compare lung HRCT analysis results with the MDA5^+^DM patients. Our study complied with the Declaration of Helsinki and informed consents were obtained from all subjects (or their legally authorized representative in cases of patient death).

### Computer-Aided Analysis of Lung HRCT

ILD include some basic radiology features in HRCT such as reticulations, septal thickening, ground glass opacities, nodules, bronchiectasis and bronchiolectasis, honeycombing, consolidation, etc. All these features showed the differences of density (CT values) in images. Our quantitative analysis method was proposed from difference of CT values in Digital Imaging and Communications in Medicine (DICOM) of HRCT images. Simply, firstly, a special lung segmentation algorithm of interstitial lung disease was developed. Then, high-precision three-dimensional images of lung interstitium were reconstructed to detect interstitial pixels beyond the limit scope of naked eyes. An index named by Effective Lung Ventilation Area Ratio (ELVAR) were calculated indicting pulmonary ventilation function.

#### Lung Segmentation

The process of lung segmentation was divided into four stages.

**Figure 2:**
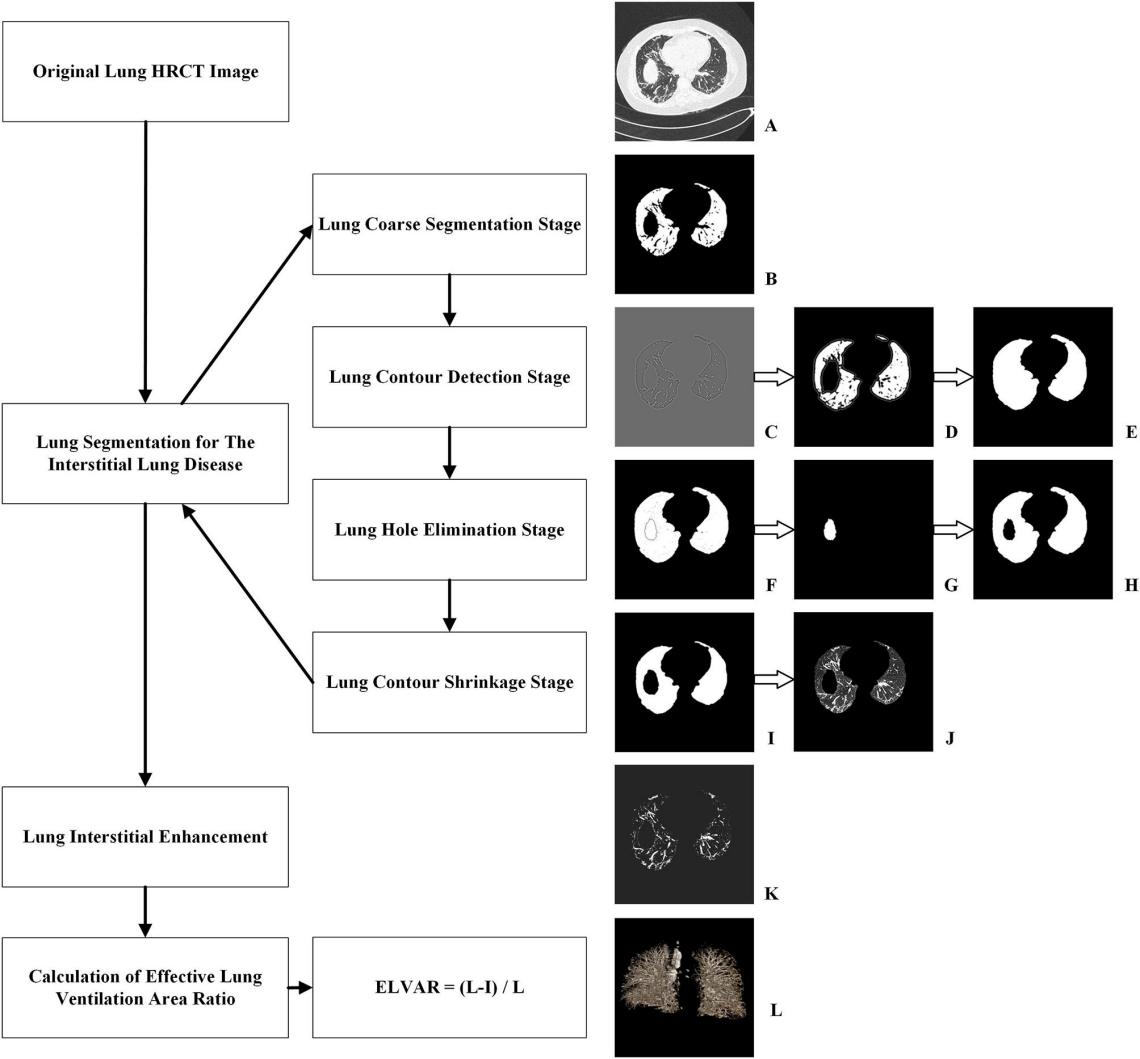
Procedure of computer-aided analysis of lung HRCT. **(A)** Original lung HRCT images were collected; **(B)** Coarse binary lung tissue region images were acquired due to difference of CT value; **(C)** Binary lung tissue region images were filtered and smoothed by Gaussian function; **(D)** Outer boundary of binary lung tissue region images was selected by Laplace operator; **(E)** The interior of lung edge was filled in binary lung tissue region images; **(F)** Images detecting hole area were superimposed onto the lung contour images; **(G)** Images with mask hole were identified; **(H)** Quasi- images of lung with mask hole were obtained by subtracting the mask hole from the lung contour image; **(I)** Images of lung masks with accurate edge were obtained; **(J)** An “and” operation produced accurate lung segmentation images; **(K)** Lung interstitial enhancement images were generated according to curvature feature of lung interstitium; **(L)** ELVAR were calculated by formula of (L–I)/L, L were the total number of lung voxels and I were the total number of interstitial voxels.

1. Lung coarse segmentation stage.
  Original lung HRCT images were collected from lung windows of every 1 mm scan (Figure 2A). Due to difference of CT value, the lung tissue can be separated with other human tissues from mediastinum to chest wall. This leaded to the generation of binary lung tissue region images (Figure 2B).
2. Lung contour detection stage.
  The task of this stage was to draw the outlines of lung based on the coarse segmentation results. Firstly, a Gaussian function was adopted to eliminate noise, hence a transition zone was produced near the lung edge, so the binary lung tissue region images were filtered and smoothed (Figure 2C). Subsequently, a Laplace operator was used to produce the inner and outer boundaries on the lung edge. The outer boundary was selected as the candidate lung edge which included all irregular fragments near the boundaries (Figure 2D). The lung contour image was finally generated by filling the interior of the candidate lung edge (Figure 2E).
3. Lung hole elimination stage.
  Because the lungs are near other organs, such as the heart, spleen, liver, and stomach, an obvious hole can appear in the CT images of the lower part of the lung. The goal of this stage was to detect and eliminate the hole area. Images detecting hole area (Figure 2D) were superimposed onto the lung contour images (Figure 2E) and were finally shown as Figure 2F. Using the region-growing method, the most lateral region of the lung was identified and set as the background, so that only the hole and blood vessels were preserved. Because blood vessels are small, the larger component with more than a specific number of pixels is regarded as the mask hole (Figure 2G). The quasi- images of lung with mask hole were obtained by subtracting the mask hole from the lung contour image(**s**).
4. Lung contour shrinkage stage.
  The main objective of this stage was to generate a more accurate lung edge. First the candidate lung edges (Figure 2E or Figure 2H) were gradually contracted until they were coincided with the zero-crossing boundary, which were regarded as the accurate edges of the lung, therefore the images of lung masks were obtained (Figure 2I). An “and” operation on the original image (Figure 2A) and its lung mask (Figure 2I) would produce accurate lung segmentation result (Figure 2J). The total number of lung voxels (L) was obtained by counting the lung pixels from accurate lung segmentation results.

#### Lung Interstitial Enhancement

Because the lung interstitium was accompanied with blood vessels, and the pixels in the pipeline significantly differed in the direction of curvature, potential lung interstitial pixels not being detected by normally vision could be detected according to their curvature feature. This allowed the identification as many interstitial pixels as possible and the lung interstitial enhancement images were generated (Figure 2K). Finally, the total number of interstitium voxels (I) was obtained by counting the pixels of all the lung interstitial enhancement images.

#### Calculation of Effective Lung Ventilation Area Ratio

**Figure 3:**
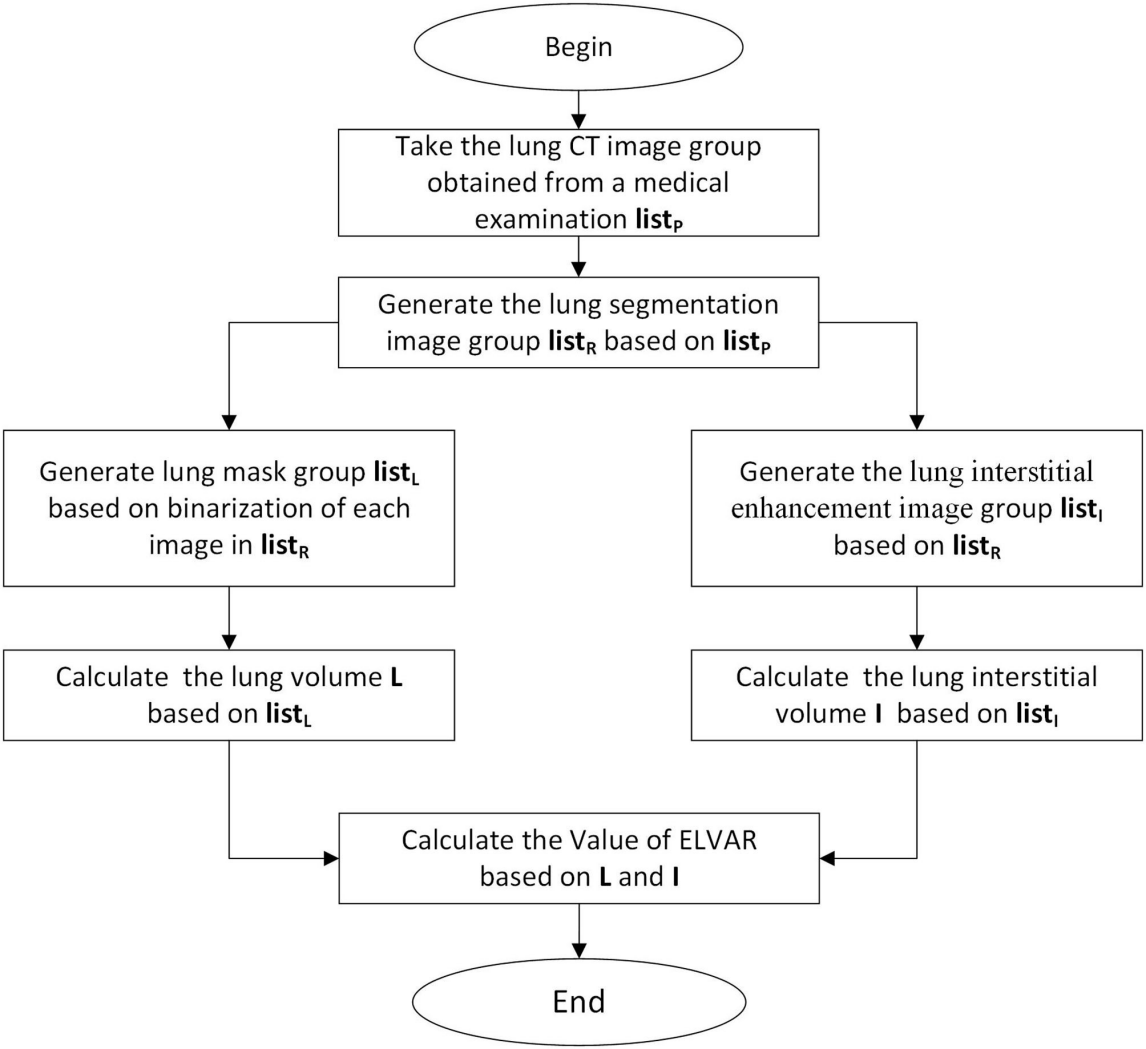
Calculation procedure of Effective Lung Ventilation Area Ratio (ELVAR). List P were original images of lung windows from HRCT **(**Figure 2A**)**. List R were segmentation results from List P **(**Figure 2J**)**. List L were data of calculation results of List R in the algorithm program **(**Figure 2I**)**. List I were data of lung interstitial enhancement of List R in the algorithm program **(**Figure 2K**)**.

**Figure 4:**
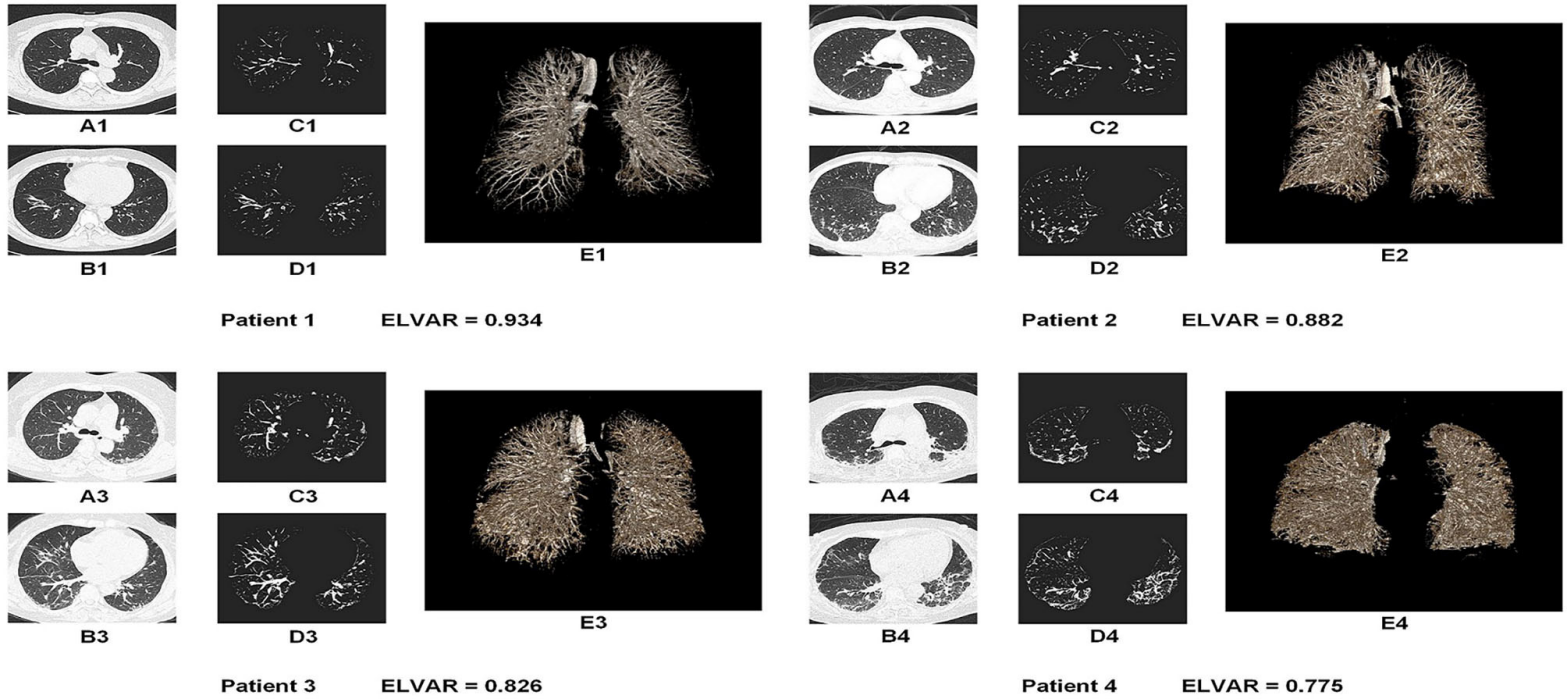
Lung images of four MDA5^+^DM patients with different value of ELVAR. **(A,B)** Original HRCT images in the different level of lung tissue (A was the section from bifurcation of the trachea and B was the section above the top of diaphragm); **(C,D)** The lung interstitial enhancement images from A and B based on computer aided analysis technique; **(E)** High precision three dimensional images of the lung interstitium from the same person.

Using L, the total number of lung voxels, and I, the total number of interstitial voxels, ELVAR can show pulmonary ventilation function by applying the formula of (L–I)/L (Figure 2L), which indirectly correlate with the scope of interstitial lung disease (Figure 3), *i.e*., low value of ELVAR means high scope of lung interstitium and low ventilation function. Using quantitative analysis method of lung segmentation and lung interstitial enhancement, the original lung HRCT images (Figures 4A1–A4,B1–B4) could be reconstructed into the lung interstitial enhancement images (Figures 4C1–C4,D1–D4) and high-precision three-dimensional images of the lung interstitium (Figures 4E1–E4), and the subtle lung interstitial changes can be more easily observed by comparing the images from different patients.

### Statistical Analysis

Statistical analysis was performed using the SPSS software version 26.0 (Chicago, IL, USA). Chi-square and Fisher's exact tests were used to compare dichotomous data, and comparisons of continuous variables were performed using Student's *t*-test or Wilcoxon's rank-sum test. Correlation analysis between ELVAR and other variables were applied with Spearman or Kendall. The receiver operator curve (ROC) analysis was performed to evaluate the diagnostic performance of the statistical significance index. Cox proportional hazards regression analysis was used to assess the prognostic factors based on Duration from treatment to death or 1 year. Odds ratios (OR) were presented with 95% confidence intervals (CI). Twelve months of survival curves of different ELVAR values of MDA5^+^DM patients were drawn using the Kaplan-Meier method. Two-tailed *p* < 0.05 were considered significant.

## Results

### Comparisons Between Early Death Group and Early Survival Group

In Table 1, the data showed that duration from onset to treatment (months) between early death group and early survival group were 2.0(1.0,3.0) and 2.5(1.5,4.0). Therefore, we had enough evidence to show that our patients were at the early stage of disease. For the 55 MDA5^+^DM patients, there were no significant difference between two groups in duration from onset to treatment and treatment drugs, which indicted the time of diagnosis and different treatment were not the risk factors leading to different prognosis. There was a significant difference in age of onset between early death group and early survival group (56[47.25,61.75] vs. 50[44,55] years), which suggested that patients with elder age onset had a higher risk of early death. Obviously, the difference among duration from treatment to death or 1 year, mortality within 3 months, mortality within 12 months between above two groups were significant. Serum C reactive protein (CRP) and level of ferritin in early death group (12.29[5.81, 23.65] mg/L and 568.35[481.71, 583.02] ng/ml, respectively) were higher than those in early survival group (4.13[2.21,11.10] mg/L and 452.07[320.44, 561.12] ng/L). However, albumin levels in early death group were lower than those in early survival group (24.40[23.50, 27.35] vs. 30.30[27.60,34.10] g/L), which indicated that MDA5^+^DM patients in early death group had aggressive systemic involvement. Other characteristics, such as the skin and muscle, laboratory data, myositis-specific autoantibody (MSA), myositis associated autoantibody (MAA) revealed no significant differences between groups (Table 1).

**Table 1:** Comparison between early death group and early survival group in MDA5^+^DM.

**Variables**	**MDA5** ^**+**^ **DM patients**		
	**Early death group *N* = 16**	**Early survival group *N* = 39**	***P*-value**
Female, n (%)	14(87.5)	28(71.8)	0.217
Age of onset (y)	56(47.25, 61.75)	50(44, 55)	0.033^*^
Duration from onset to treatment, m	2.0(1.0, 3.0)	2.5(1.5, 4.0)	0.315
Duration from treatment to death or 1 year, m	0.75(0.5, 1.5)	12(12.0, 12.0)	0.000^*^
Mortality within 3 months, n (%)	16(100)	0 (0)	0.000^*^
Mortality within 12 months, n (%)	16(100)	1(2.6)	0.000^*^
Smoking history, n (%)	4(25.0)	4(10.3)	0.163
**Clinical feature**			
Muscle weakness, n (%)	8(50.0)	25(64.1)	0.337
Gottron sign, n (%)	5(31.3)	9(23.1)	0.531
Heliotrop rash, n (%)	4(25.0)	15(38.5)	0.345
Skin ulcer, n (%)	2(12.5)	12(30.8)	0.162
Raynaud phenomenon, n (%)	0(0.0)	3(7.7)	0.258
Fever, n (%)	7(43.8)	8(20.5)	0.082
Cough, n (%)	2(12.5)	9(23.1)	0.377
Dyspnea, n (%)	12(75.0)	22(56.4)	0.202
Pneumothorax, n (%)	1(6.3)	1(2.6)	0.511
Concomitant neoplasia, n (%)	0(0.0)	1(2.6)	0.522
Laboratory data			
Neutrophil Count, X10^9^/L	3.31(2.57, 4.59)	3.29(2.43, 4.86)	0.978
Lymphocyte Count, X10^9^/L	0.86(0.45, 1.14)	0.85(0.56, 1.21)	0.670
ESR, mm/hr	43(25, 61)	38.5(28.5, 56.25)	1.000
CRP, mg/L	12.29(5.81, 23.65)	4.13(2.21, 11.10)	0.026^*^
CK, IU/L	80.10(49.30, 144.23)	84.70(41.50, 191.40)	0.704
LDH, IU/L	382.55(351.30, 535.40)	364.30(266.90, 445.40)	0.069
Myoglobin, ug/L	101(57.65, 111.35)	66.20(38.50, 95.20)	0.132
Albumin, g/L	24.40(23.50, 27.35)	30.30(27.60, 34.10)	0.000^*^
Ferritin, ng/ml	568.35(481.71, 583.02)	452.07(320.44, 561.12)	0.001^*^
PCT, ng/ml	0.11(0.053, 0.193)	0.07(0.04, 0.105)	0.089
Pulmonary involvement			
ILD diagnosis, n (%)^&^	16(100)	32(82.1)	0.072
Feature of ILD at diagnosis			
Reticulations, n (%)	3(18.75)	11(28.21)	0.469
Septal thickening, n (%)	8(50.00)	11(28.21)	0.126
Ground glass opacities, n (%)	4(25.00)	9(23.08)	0.880
Nodules, n (%)	5(31.25)	7(17.95)	0.282
Bronchiectasis/Bronchiolectasis, n (%)	1(6.25)	2(5.13)	0.869
Honeycombing, n (%)	1(6.25)	1(2.56)	0.511
Consolidation, n (%)	12(75.00)	22(56.41)	0.202
ELVAR	0.7973(0.7458, 0.8338)	0.8685(0.8155, 0.8956)	0.001^*^
Severity of type I respiratory failure			
no/mild/moderate/severe, n (%)	4/3/7/2 (25.0/18.8/43.7/12.5)	31/5/3/0 (79.5/12.8/7.7/0.0)	0.000^*^
no, n (%)	4(25.0)	31(79.5)	0.000^*^
mild, n (%)	3(18.8)	5(12.8)	0.575
moderate, n (%)	7(43.7)	3(7.7)	0.002^*^
severe, n (%)	2(12.5)	0(0)	0.026^*^
P/F ratio, mmHg	226(188, 297.75)	417(312, 476)	0.000^*^
Oxygen supplementation			
0/1-2/3-4/>4, L/min, n (%)	3/1/6/6 (18.8/6.2/37.5/37.5)	19/8/9/3 (48.7/20.5/23.1/7.7)	0.003^*^
0L/min, n (%)	3(18.8)	19(48.7)	0.040^*^
1–2L/min, n (%)	1(6.2)	8(20.5)	0.198
3–4L/min, n (%)	6(37.5)	9(23.1)	0.280
>4L/min, n (%)	6(37.5)	3(7.7)	0.007^*^
FVC, % PRED	42(37, 53)	79(78, 81)	0.004^*^
DLCO, % PRED	35(28, 46)	79(78, 80)	0.004^*^
Antibody positive			
ANA, n (%)	2(12.5)	15(38.5)	0.061
MAA profile	14(87.5)	27(69.2)	0.162
SSA, n (%)	0(0.0)	8(20.5)	0.052
SSB, n (%)	0(0.0)	3(7.7)	0.258
Ro-52, n (%)	14(87.5)	25(64.1)	0.086
CENP-B, n (%)	1(6.3)	1(2.6)	0.511
Ku, n (%)	0(0.0)	1(2.6)	0.522
PM-Scl, n (%)	0(0.0)	2(5.1)	0.361
MSA profile, n (%)	5(31.3)	8(20.5)	0.399
anti-ARS antibodies, n (%)	1(6.3)	4(10.3)	0.642
Jo-1, n (%)	0(0.0)	2(5.1)	0.361
PL-7, n (%)	0(0.0)	1(2.6)	0.522
PL-12, n (%)	0(0.0)	2(5.1)	0.361
EJ, n (%)	0(0.0)	0(0.0)	1.000
OJ, n (%)	1(6.3)	0(0.0)	0.118
Non anti ARS antibodies, n (%)	4(25.0)	6(15.4)	0.405
SRP, n (%)	0(0.0)	2(5.1)	0.361
TIF1-γ, n (%)	1(6.3)	2(5.1)	0.869
Mi-2β, n (%)	3(18.8)	2(5.1)	0.114
SAE, n (%)	0(0.0)	1(2.6)	0.522
Treatment			
Glucocorticoid, <1 /≥1 mg/kg/d, n (%)	6/10(37.5/62.5)	22/17(56.4, 43.6)	0.207
Methotrexate, n (%)	0(0.0)	5(12.8)	0.137
Cyclophosphamide, n (%)	12(75.0)	27(69.2)	0.672
Cyclosporine A, n (%)	1(6.3)	7(17.9)	0.268
Tacrolimus, n (%)	1(6.3)	6(15.4)	0.360
Plasma exchange, n (%)	4(25.0)	3(7.7)	0.083
Single drug or Combination drugs			
1 drug	2(12.5)	6(15.4)	0.785
2 drugs	11(68.8)	20(51.3)	0.240
3 drugs	2(12.5)	11(28.2)	0.217
4 drugs	1(6.3)	2(5.1)	0.869

* *P < 0.05*.

& *ILD diagnosis by a radiologist and a respirologist simultaneous*.

*ANA, antinuclear antibody; CRP, C reactive protein; CK, creatine kinase; DLCO, carbon monoxide diffusing capacity; ELVAR, Efficient Lung Vetilation Area Ratio; ESR, erythrocyte sedimentation rate; FVC, forced vital capacity; ILD, interstitial lung disease; LDH, Lactic dehydrogenase; MDA5, melanoma melanoma differentiation associated gene 5; PCT, procalcitonin; P/F ratio, PaO2/FiO2 ratio; SAE, anti-small ubiquitin-like modifier antibody; SRP, signal recognition particle; TIF1-γ, transcription intermediary factor 1-gamma*.

**Figure 5:**
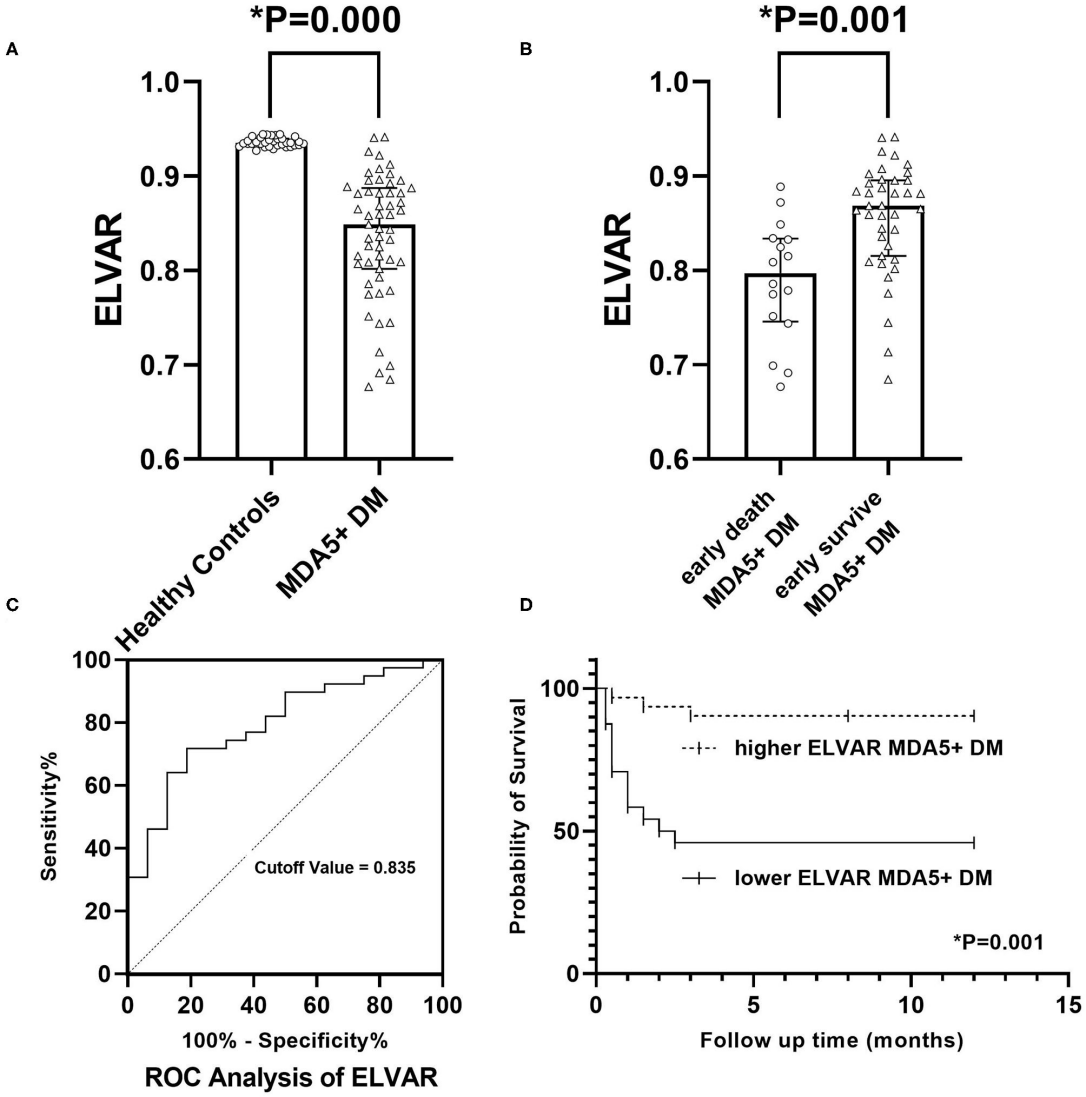
Clinical significance of value of ELVA. **(A)** Comparison of ELVAR values between healthy controls and MDA5^+^DM patients; **(B)** Comparison of ELVAR values between early death MDA5^+^DM patients and early survival MDA5^+^DM patients; **(C)** ROC analysis of ELVAR and best cutoff value was 0.835; **(D)** Comparison of 12-month Kaplan-Meier survival curve between higher ELVAR MDA5^+^DM patients and lower ELVAR MDA5^+^DM patients.

For pulmonary involvement, there were no significant differences in cough, pneumothorax, ILD diagnosis, or radiological features of ILD. The values of ELVAR indirectly reflecting the severity of interstitial structure were significantly different between early death group and early survival group (0.7973[0.7458, 0.8338] vs. 0.8685[0.8155, 0.8956], Figure 5B). The differences among severity of type I respiratory failure at diagnosis (no, mild and severe), P/F ratio and oxygen supplementation (0 and >4 L/min) between two groups were significantly. The two indices of the lung function test, FVC % predicted (FVC PRED) and DLCO% predicted (DLCO%PRED), were 42 (37, 53) and 35 (28, 46)%, respectively, in early death group, which were lower than those in early survival group (79 [78, 81] and 79 [78, 80]%).

### ROC Analysis and Correlation Analysis

The ROC analysis of the eight significantly different indices (i.e., age at onset, CRP, level of ferritin, value of ELVAR, P/F ratio, level of albumin, FVC and DLCO) showed that value of ELVAR, level of albumin and P/F ratio had well diagnostic value for early death within 3 months with higher sensitivity (71.8, 87.2, and 87.2%) and specificity (81.2, 75.0, and 75.0%). FVC and DLCO with lower specificity will be verified with more data in the future because only two patients underwent a lung function test and the remaining 14 patients did not accept the lung function test because of intolerability or early death in the early death group of the study (Table 2). As for correlation analysis, level of albumin, value of ELVAR and P/F ratio had different correlation with all indices except for FVC and DLCO, the scope of correlation about value of ELVAR lied in moderate range (Table 3). ROC analysis and correlation analysis indicted value of ELVAR had good diagnostic and correlation with clinical characteristics.

**Table 2:** Analysis of ROC by eight significantly different indices between early death group and early survival group.

	**Analysis of ROC**				
	**AUC**	**Value of cut-off**	**Sensitivity (%) (C.I.)**	**Specificity (%) (C.I.)**	***p* value**
Age at onset	0.684	54.5	56.3	74.4	0.033^*^
CRP	0.692	8.51	68.8	74.4	0.026^*^
Ferritin	0.776	568.34	53.8	96.9	0.004^*^
ELVAR	0.795	0.835	71.8	81.2	0.001^*^
P/F ratio	0.822	269.5	87.2	75.0	0.000^*^
Albumin	0.829	26.65	87.2	75.0	0.000^*^
FVC^#^	1.000	64.5	100.0	0.0	0.004^*^
DLCO^#^	1.000	61.0	100.0	0.0	0.004^*^

* *P < 0.05*.

# *Only two patients in early death group accepted lung function test and other 14 patients in early death group didn't accept lung function test for rapidly death or intolerable*.

*CRP, C reactive protein; DLCO, carbon monoxide diffusing capacity; ELVAR, Efficient Lung Ventilation Area Ratio; FVC, forced vital capacity; P/F ratio, PaO2/FiO2 ratio*.

**Table 3:** Correlation analysis between value of ELVAR, level of Albumin, P/F ration and significant different indices from comparison between early death group and early survival group.

**Correlation analysis**	**Value of ELVAR**				**Level of Albumin**				**P/F ratio**			
	**Spearman ρ**	***P-*value**	**Kendall τ**	***P-*value**	**Spearmanρ**	***P-* value**	**Kendallτ**	***P-* value**	**Spearman ρ**	***P-*value**	**Kendall τ**	***P-*value**
Duration from treatment to death or 1 year	0.488	0.000^*^			0.502	0.000^*^			0.511	0.000^*^		
ELVAR	/	/			0.363	0.006^*^			0.323	0.016^*^		
Albumin	0.363	0.006^*^			/	/			0.433	0.001^*^		
P/F ratio	0.323	0.016^*^			0.433	0.001^*^			/	/		
Ferritin	−0.295	0.029^*^			−0.391	0.003^*^			−0.245	0.072		
Age at onset	−0.247	0.069			−0.356	0.008^*^			−0.211	0.121		
CRP	−0.219	0.108			−0.455	0.000^*^			−0.322	0.017^*^		
FVC	−0.010	0.953			−0.046	0.785			0.029	0.865		
DLCO	−0.015	0.928			−0.197	0.236			0.286	0.082		
Mortality within 3 months			0.382	0.001^*^			0.428	0.000^*^			0.419	0.000^*^
Mortality within 12 months			0.363	0.001^*^			0.429	0.000^*^			0.399	0.000^*^
Oxygen supplementation			−0.287	0.006^*^			−0.219	0.035^*^			−0.658	0.000^*^
Severity of type I respiratory failure			−0.287	0.007^*^			−0.301	0.005^*^			−0.725	0.000^*^

* *P < 0.05*.

*CRP, C reactive protein; DLCO, carbon monoxide diffusing capacity; ELVAR, Efficient Lung Vetilation Area Ratio; FVC, forced vital capacity; P/F ratio, PaO2/FiO2 ratio*.

### Clinical Significance of ELVAR Values

#### Distribution and Comparison of ELVAR Values Between Healthy Controls and MDA5^+^DM Patients

Using computer-assisted lung HRCT image analysis, we found that the lungs of MDA5^+^DM patients differed significantly from those of healthy controls. There were interstitial hyperplasia structures in the outer lung area of patients. In severe cases, it had spread to the middle and upper regions of the lung (Figure 4). Results showed that value of ELVAR in healthy controls were higher than those in MDA5^+^DM patients (0.9351[0.9311, 0.9414] vs. 0.8487[0.8018, 0.8875]; Figure 5A).

#### Difference Between the Lower ELVAR Group and Higher ELVAR Group

The ROC analysis of ELVAR values showed that the best diagnostic cutoff value was 0.835 (Figure 5C). Based on this ELVAR cutoff value, 24 MDA5^+^DM patients were classified into the lower ELVAR group (ELVAR <0.835) and 31 into the higher ELVAR group (ELVAR ≥ 0.835). The comparison revealed that the rates of mortality within 3 months in the lower and higher ELVAR groups were 54.2 vs. 9.7%, respectively, whereas 12-month mortality rates were 54.2 vs. 12.9%, respectively (Figure 5D), which indicated that the ELVAR value was more predictive of early mortality within 3 months. The differences among severity of type I respiratory failure at diagnosis (no), P/F ratio and oxygen supplementation (0 and >4 L/min) between two groups were significantly. For the other indices, dyspnea in lower ELVAR group was more frequent than that in higher ELVAR group (79.2 vs. 48.4%), and lymphocyte count of lower ELVAR group was lower than that of higher ELVAR group (0.65[0.50, 0.87] vs. 0.92[0.63, 1.34] × 10^9^/L; Table 4).

**Table 4:** Comparison between lower ELVAR group and higher ELVAR group.

**Variables**	**MDA5** ^**+**^ **DM patients**		
	**lower ELVAR group *N* = 24**	**higher ELVAR group *N* = 31**	***P*-value**
Female, n (%)	20(83.3)	22(71.0)	0.289
Age of onset (y)	53(46.25, 59.25)	52(42, 59)	0.440
Duration from onset to treatment, m	3.0(2.0, 4.0)	2.0(1.0, 3.0)	0.095
Duration from treatment to death or 1 year, m	2.25(0.5, 12.0)	12.0(12.0, 12.0)	0.000^*^
Mortality within 3 months, n (%)	13(54.2)	3(9.7)	0.000^*^
Mortality within 12 months, n (%)	13(54.2)	4(12.9)	0.001^*^
Smoking history, n (%)	3(12.5)	5(16.1)	0.708
Clinical feature			
Muscle weakness, n (%)	12(50.0)	21(67.7)	0.187
Gottron sign, n (%)	7(29.2)	7(22.6)	0.582
Heliotrop rash, n (%)	8(33.3)	11(35.5)	0.869
Skin ulcer, n (%)	6(25.0)	8(25.8)	0.946
Raynaud phenomenon, n (%)	0(0.0)	3(9.7)	0.120
Fever, n (%)	8(33.3)	7(22.6)	0.379
Cough, n (%)	4(16.7)	7(22.6)	0.590
Dyspnea, n (%)	19(79.2)	15(48.4)	0.021^*^
Pneumothorax, n (%)	1(4.1)	1(3.2)	0.855
Concomitant neoplasia, n (%)	0(0.0)	1(2.6)	0.379
Laboratory data			
Neutrophil Count, X10^9^/L	3.31(2.67, 4.74)	3.29(2.37, 4.63)	0.754
Lymphocyte Count, X10^9^/L	0.65(0.50, 0.87)	0.92(0.63, 1.34)	0.022^*^
ESR, mm/hr	43(31, 62)	32.50(24.50, 56.25)	0.164
CRP, mg/L	9.21(2.60, 18.03)	5.16(2.60, 11.10)	0.222
CK, IU/L	76.20(45.83, 144.23)	87.4(44.6, 201.2)	0.905
LDH, IU/L	378.60(332.80, 525.55)	358.50(268.25, 441.05)	0.157
Myoglobin, ug/L	69.60(49.50, 108.78)	69.4(38.5, 95.2)	0.457
Albumin, g/L	27.40(24.33, 30.83)	29.8(26.7, 34.2)	0.051
Ferritin, ng/ml	519.05(375.45, 570.19)	485.18(357.90, 562.67)	0.131
PCT, ng/ml	0.09(0.053, 0.14)	0.07(0.035, 0.11)	0.214
Pulmonary involvement			
ILD diagnosis, n (%)^&^	22(91.7)	26(83.9)	0.394
Radiological feature of ILD at diagnosis			
Reticulations, n (%)	6(25.0)	8(25.8)	0.946
Septal thickening, n (%)	8(33.3)	11(35.5)	0.869
Ground glass opacities, n (%)	7(29.2)	6(19.4)	0.400
Nodules, n (%)	3(12.5)	9(29.0)	0.145
Bronchiectasis/Bronchiolectasis, n (%)	1(4.2)	2(6.5)	0.714
Honeycombing, n (%)	0(0.0)	2(6.5)	0.209
Consolidation, n (%)	18(75.0)	16(51.6)	0.079
ELVAR	0.7893(0.7442,0.8143)	0.8823(0.8638,0.9025)	0.000^*^
Severity of type I respiratory failure			
no/mild/moderate/severe, n (%)	11/4/7/2 (45.8/16.7/29.2/8.3)	24/4/3/0 (77.4/12.9/9.7/0)	0.009^*^
no, n (%)	11(45.8)	24(77.4)	0.017^*^
mild, n (%)	4(16.7)	4(12.9)	0.697
moderate, n (%)	7(29.2)	3(9.7)	0.066
severe, n (%)	2(8.3)	0(0)	0.105
P/F ratio, mmHg	260(192.5, 419)	417(303, 471)	0.025^*^
Oxygen supplementation			
0/1–2/3–4/>4L/min, n (%)	5/5/7/7 (20.8/20.8/29.2/29.2)	17/4/8/2 (54.8/12.9/25.8/6.5)	0.007^*^
0L/min, n (%)	5(20.8)	17(54.8)	0.011^*^
1–2L/min, n (%)	5(20.8)	4(12.9)	0.435
3–4L/min, n (%)	7(29.2)	8(25.8)	0.783
>4L/min, n (%)	7(29.2)	2(6.5)	0.025^*^
FVC, % PRED	79(71,82)	79(78,81)	0.843
DLCO, % PRED	79(77.25,79.75)	79(68.50,80.25)	0.902
Antibody positive			
ANA, n (%)	7(29.2)	10(32.3)	0.807
MAA profile	19(79.2)	22(71.0)	0.493
SSA, n (%)	2(8.3)	6(19.4)	0.255
SSB, n (%)	2(8.3)	1(3.2)	0.412
Ro-52, n (%)	18(75.0)	21(67.7)	0.560
CENP-B, n (%)	1(4.2)	1(3.2)	0.855
Ku, n (%)	0(0.0)	1(3.2)	0.379
PM-Scl, n (%)	2(8.3)	0(0.0)	0.105
MSA profile, n (%)	7(29.2)	6(19.4)	0.400
anti-ARS antibodies, n (%)	2(8.3)	3(9.7)	0.865
Jo-1, n (%)	0(0.0)	2(6.5)	0.209
PL-7, n (%)	0(0.0)	1(3.2)	0.379
PL-12, n (%)	1(4.2)	1(3.2)	0.855
EJ, n (%)	0(0.0)	0(0.0)	1.000
OJ, n (%)	1(4.2)	0(0.0)	0.256
Non-anti-ARS antibodies, n (%)	5(20.8)	5(16.1)	0.657
SRP, n (%)	1(4.2)	1(3.2)	0.855
TIF1-γ, n (%)	1(4.2)	2(6.5)	0.714
Mi-2β, n (%)	2(8.3)	3(9.7)	0.865
SAE, n (%)	1(4.2)	0(0.0)	0.256
Treatment			
Glucocorticoid, <1 /≥1 mg/kg/d, n (%)	12/12(50.0/50.0)	16/15(51.6/48.4)	0.906
Methotrexate, n (%)	2(8.3)	3(9.7)	0.865
Cyclophosphamide, n (%)	19(79.2)	20(64.5)	0.240
Cyclosporine A, n (%)	1(4.2)	7(22.6)	0.057
Tacrolimus, n (%)	2(8.3)	5(16.1)	0.394
Plasma exchange, n (%)	4(16.7)	3(9.7)	0.445
Single drug or Combination drugs			
1 drug	3(12.5)	5(16.1)	0.708
2 drugs	15(62.5)	16(51.6)	0.424
3 drugs	5(20.8)	8(25.8)	0.670
≥ 4 drugs	1(4.2)	2(6.5)	0.714

* *P < 0.05*.

& *ILD diagnosis by a radiologist and a respirologist simultaneous*.

*ANA, antinuclear antibody; CRP, C reactive protein; CK, creatine kinase; DLCO, carbon monoxide diffusing capacity; ELVAR, Efficient Lung Vetilation Area Ratio; ESR, erythrocyte sedimentation rate; FVC, forced vital capacity; ILD, interstitial lung disease; LDH, Lactic dehydrogenase; MDA5, melanoma melanoma differentiation-associated gene-5; PCT, procalcitonin; P/F ratio, PaO2/FiO2 ratio; SAE, anti-small ubiquitin-like modifier antibody; SRP, signal recognition particle; TIF1-γ, transcription intermediary factor 1-gamma*.

#### ELVAR as a Risk Factor for Poor Prognosis of MDA5^+^DM Patients

To identify the indicators of poor outcomes in MDA5^+^DM patients, we performed Cox regression analysis. Results of the univariate analysis revealed that age of onset, CRP, myoglobin, albumin, ferritin, PCT, ELVAR, severity of type I respiratory failure, P/F ratio, oxygen supplementation, FVC and DLCO were significantly associated with poor outcomes. Only four factors including age of onset, ferritin, value of ELVAR and oxygen supplementation >4 L/min showed significantly value for poor prognosis in MDA5^+^DM patients in the multivariate analysis (Table 5). FVC and DLCO were excluded from the multivariate analysis because of insufficient data due to patient intolerability and early-death patients.

**Table 5:** Univariate analysis and Multivariate analysis of prognostic factors in MDA5^+^DM patients.

**Variable**	**Univariate analysis**		**Multivariate analysis**	
	**HR (95%CI)**	***P-* value**	**HR (95%CI)**	***P-* value**
Female	0.420(0.095–1.852)	0.252		
Age at onset	2.776(1.031–7.471)	0.043^*^	9.842(1.434–67.528)	0.020^*^
Duration from onset to treatment	0.550(0.200–1.515)	0.247		
Mortality within 3 months	0.000(0.000–1.845)	0.069		
Mortality within 12 months	0.001(0.000–1.294)	0.059		
Smoking history	2.309(0.743–7.171)	0.148		
Muscle weakness	0.616(0.231–1.644)	0.334		
Gottron sign	1.311(0.455–3.776)	0.616		
Heliotrop rash	0.598(0.193–1.856)	0.374		
Skin ulcer	0.384(0.087–1.689)	0.205		
Raynaud phenomenon	0.045(0.000–351.921)	0.498		
Fever	2.336(0.869–6.282)	0.093		
Cough	0.527(0.120–2.321)	0.397		
Dyspnea	2.254(0.725–7.002)	0.160		
Pneumothorax	2.142(0.282–16.282)	0.462		
Concomitant neoplasia	0.048(0.000–216181.008)	0.698		
Neutrophil Count	0.272(0.036–2.058)	0.207		
Lymphocyte Count	0.448(0.163–1.237)	0.121		
ESR	0.328(0.093–1.156)	0.083		
CRP	4.665(1.615–13.474)	0.004^*^	2.172(0.480–9.841)	0.314
CK	5.104(0.674–38.674)	0.115		
LDH	36.822(0.430–3154.017.039)	0.112		
Myoglobin	3.004(1.124–8.031)	0.028^*^	3.347(0.783–14.306)	0.103
Albumin	0.109(0.035–0.342)	0.000^*^	0.307(0.053–S1.785)	0.189
Ferritin	7.897(2.862–21.793)	0.000^*^	11.255(2.084–60.786)	0.005^*^
PCT	2.793(1.012–7.707)	0.047^*^	0.954(0.217–4.187)	0.950
ILD diagnosis	25.672(0.061–10818.508)	0.293		
Reticulations	0.630(0.179–2.210)	0.470		
Septal thickening	2.228(0.835–5.940)	0.109		
Ground glass opacities	1.202(0.388–3.730)	0.750		
Nodules	1.542(0.535–4.440)	0.422		
Bronchiectasis/Bronchiolectasis	1.413(0.186–10.712)	0.738		
Honeycombing	2.142(0.282–16.282)	0.462		
Consolidation	2.081(0.671–6.454)	0.205		
ELVAR	0.133(0.038–0.468)	0.002^*^	0.098(0.017–0.564)	0.009^*^
Severity of type I respiratory failure				
Mild	7.399(2.370–23.106)	0.001^*^	0.001(0.000–4.093E+100)	0.995
Moderate	8.453(3.075–23.234)	0.000^*^	5.369(0.676–42.654)	0.112
Severe	8.516(1.719–42.197)	0.009^*^	1.062(0.088–12.815)	0.962
P/F ratio	0.095(0.030–0.298)	0.000^*^	0.000(0.000–4.865E+99)	0.941
Oxygen supplementation				
1–2	3.370(0.958–11.853)	0.058		
3–4	4.802(1.542–14.954)	0.007^*^	3.476(0.286–42.194)	0.328
>4	3.711(1.337–10.299)	0.012^*^	0.010(0.001–0.146)	0.001^*^
FVC	1.000	1.000	NC^&^	
DLCO	1.000	1.000	NC^&^	
ANA	0.296(0.067–1.303)	0.107		
SSA	0.037(0.000–11.180)	0.259		
SSB	0.045(0.000–351.921)	0.498		
RO-52	2.985(0.677–13.150)	0.148		
CENP-B	2.142(0.282–16.282)	0.462		
Ku	0.048(0.000–216181.008)	0.698		
PM-Scl	0.047(0.000–2532.428)	0.582		
Jo-1	0.047(0.000–2532.428)	0.582		
PL-7	0.048(0.000–216181.008)	0.698		
PL-12	0.047(0.000–2532.428)	0.582		
OJ	4.085(0.531–31.432)	0.176		
SRP	0.047(0.000–2532.428)	0.582		
TIF1-γ	1.239(0.164–9.388)	0.836		
Mi-2β	2.196(0.625–7.713)	0.220		
SAE	0.048(0.000–216181.008)	0.698		
Glucocorticoid	1.831(0.665–5.043)	0.242		
Methotrexate	0.042(0.000–48.056)	0.378		
Cyclophosphamide	1.176(0.379–3.648)	0.779		
Cyclosporine A	0.363(0.048–2.747)	0.326		
Tacrolimus	0.415(0.055–3.143)	0.395		
Plasma exchange	2.812(0.900–8.787)	0.075		
Single drug or Combination drugs				
1 drug	0.894(0.203–3.935)	0.882		
2 drugs	1.119(0.254–4.923)	0.882		
3 drugs	0.514(0.146–1.804)	0.299		
≥4 drugs	239(0.164–9.388)	0.836		

* *P < 0.05*.

& *NC, only 2 patients in early death group accepted lung function test and other 14 patients in early death group didn't accept lung function test for rapidly death or intolerable. Hence multivariate analysis didn't include the FVC and DLCO into analysis*.

## Discussion

It is widely known that the short-term prognosis of MDA5^+^DM-ILD patients is poor (18). Accurate prediction of MDA5^+^DM patient outcomes is a key issue in clinical practice. In this study, we established a computer-aided lung interstitial image analysis approach. ELVAR was obtained by calculating the proportion of areas outside the lung interstitium in lung tissue that can indirectly evaluates the severity of lung interstitial lesions. Our results showed that ELVAR values in MDA5^+^DM patients who died within 3 months after diagnosis were significantly reduced than those who were survival after 3 months. ROC analysis and correlation analysis about value of ELVAR showed that ELVAR values have good predictive power for 3-month mortality and widely correlation with many clinical indices, which indicates that it may be a powerful tool for clinicians to evaluate poor outcomes in MDA5^+^DM-ILD.

Recently a variety of lung segmentation methods have been advanced (19). According to the different segmentation features used, lung segmentation methods can be divided into threshold-based methods (20), region-based methods (21), and shape-based methods (22). However, these methods had different degrees of partial lung tissue loss. A recent study used deep learning and feature learning to obtain better segmentation results by training with large samples (23). Software package named “CT Pneumonia Analysis” applying in the similar mechanism was used in study by S Ye recently (24). Our design idea was to extent different. We considered deep learning above cannot guarantee the accuracy results for inconsistent diagnosis of complex images and was not feasible for rare diseases such as MDA5^+^DM. We proposed another lung tissue segmentation method that was suitable for CT images of interstitial lung disease. The lung edges produced by our method were more accurate than those of above mentioned traditional methods (25). The outstanding feature lying in our computer-aided analysis of lung HRCT were outer boundary of binary lung tissue region images selected by Laplace operator and interstitial enhancement generated according to curvature feature of lung interstitium. Because our imagine analysis had based on high density structure from HRCT, we can apprehension that imagine of normal lung interstitium and abnormal imagine including consolidation, reticulations, septal thickening, ground glass opacities, nodules, bronchiectasis, honey combing, cysts could be detected and analysis (26). Because ELVAR was defined with the formula of (L–I)/L (L was the total number of lung voxels and I was the total number of interstitial voxels), which could easily be understood that the level of ELVAR was negative correlation with the scope of abnormal lung interstitium, *i.e*., high value of ELVAR meant less scope of interstitium and high ventilation, however, the lower value of ELVAR referred to large scope of interstitium and low ventilation which possible indicted poor disease outcome. Finally in our single-center retrospective study, we calculated that value of ELVAR in healthy controls was 0.938 while cut off value of ELVAR by ROC analysis in MDA5^+^DM-ILD was 0.835. Patients with low ELVAR values (<0.835) had a 54.2% chance of dying within 3 months, which may be valuable for clinicians to administer more active treatments in these patients.

The prediction value of the pulmonary function test could not be evaluated in this study because 14 patients in early death group did not accept the lung function test for early death or intolerability. In terms of albumin levels, it is widely accepted that patients with hypoalbuminemia do not recover easily; however, there is currently no data to directly verify the relationship between albumin level and prognosis of patients with MDA5^+^DM. In 2020, a prediction model using CRP and Krebs Von den Lungen-6 (KL-6) combined with the anti-MDA5 antibody was shown to be useful for predicting prognosis of patients with DM-ILD (27). As for ferritin, many studies have shown that serum ferritin levels were not only related to the severity of interstitial pneumonia in patients (28) but also predicted the risk of death (29). Recently, a FLAIR model was developed that combined five clinical indices, which included ferritin, lactate dehydrogenase, MDA5 antibody, HRCT Imagine semi-quantitative score, and rapidly-progressive ILD, to predict amyopathic dermatomyositis survival time (30). This FLAIR model was a multivariable model with rigorously established cutoff values that can be applied by clinicians in a uniform way. The 1-year survival in the discovery and validation cohorts were 34.3% and 15.0% for the high-risk group. This will provide a guidepost for making decisions around how aggressively to immunosuppress (31). Our study showed four factors including age of onset, ferritin, value of ELVAR and oxygen supplementation >4 L/min predicted significantly value for poor prognosis in MDA5^+^DM patients, which was consistent with FLAIR model to some extent. Above all, these findings highlight the value and importance of quantitative lung involvement of ILD. Our research based on computer-based image analysis technology that attempt to directly quantify lung interstitial lesions, which was simpler and more quantitative than FLAIR's multi-index model, and had a certain degree of innovation.

As an exploratory study that combined computer image analysis and prognosis of MDA5^+^DM, several limitations may be noticed. First, this was a single-center study, and more data from multi-centers are needed to confirm the efficiency of ELVAR values. Second, the role of ELVAR needs to be verified among different diseases' associated ILD. Third, identification will be needed regarding whether computer-aided analysis of lung HRCT based on Siemens system can be applied to GE or Samsung system.

## Conclusion

An index named by ELVAR basing on lung segmentation and lung interstitial enhancement was successfully established. The study aimed to assess the role of ELVAR value in predicting early death from interstitial pneumonia secondary to MDA5^+^DM. A retrospective analysis of 46 healthy controls and 55 patients with MDA5^+^DM from a single center found that the ELVAR value of healthy controls was stable at around 0.938, whereas MDA5^+^DM patients had lower values. Results showed that early mortality within 3 months of onset in patients with ELVAR values lower than 0.835 was significantly high as 54.2%. This novel index may be valuable for clinicians to efficiently identify severe patients and administer more active treatments.

## Data Availability Statement

The raw data supporting the conclusions of this article will be made available by the authors, without undue reservation.

## Ethics Statement

The studies involving human participants were reviewed and approved by Ethics Committee of Second Xiangya Hospital of Central South University. The patients/participants provided their written informed consent to participate in this study. Written informed consent was obtained from the individual(s) for the publication of any potentially identifiable images or data included in this article.

## Author Contributions

CW, XM, and FeL designed the research. CW, LG, and FaL developed the computer-aided image analysis technology. XM and HL evaluated the ILD diagnosis of MDA5^+^DM. CW, JD, and FeL were responsible for the statistical analysis. CW and FeL wrote the paper. All authors contributed to the article and approved the submitted version.

## Funding

National foundation of HUNAN province (2021JJ30934); Educational Fund of Hunan Provincial Finance Department (2021-22-2050205); 225 Talent Project of Hunan Provincial Health Committee (2019-196); and Project of Changsha Science and Technology Bureau (kq1901119).

## Conflict of Interest

The authors declare that the research was conducted in the absence of any commercial or financial relationships that could be construed as a potential conflict of interest.

## Publisher's Note

All claims expressed in this article are solely those of the authors and do not necessarily represent those of their affiliated organizations, or those of the publisher, the editors and the reviewers. Any product that may be evaluated in this article, or claim that may be made by its manufacturer, is not guaranteed or endorsed by the publisher.
